# Successful Treatment of Subacute Thyroiditis After Recombinant COVID-19 Vaccination Using Traditional Chinese Medicine: A Case Report

**DOI:** 10.7759/cureus.30716

**Published:** 2022-10-26

**Authors:** Yong Zhao, Bo Zhao, Ying bing Liang

**Affiliations:** 1 Respiratory Medicine, The Traditional Chinese Medicine Hospital of Lang fang City, LangFang City, CHN

**Keywords:** glucocorticoid, traditional chinese medicine, vaccination, sars-cov-2, subacute thyroiditis

## Abstract

Subacute thyroiditis (SAT) is a thyroid inflammatory disease, which can be triggered by viral infection. The severe acute respiratory syndrome-coronavirus-2 (SARS-CoV-2) is considered to be a potent SAT-triggering factor in this COVID-19 pandemic. However, SAT occurring after SARS-CoV-2 vaccination is rarely reported. Despite the high availability of diagnostic tools, the recurrence and steroid dependence as well as delayed diagnosis of SAT remain. This paper reports a rare case where a patient was diagnosed with SAT post receiving a recombinant novel coronavirus vaccine (Anhui Zhifei Longcom Biopharmaceutical Co.Ltd., China), and efficiently treated with traditional Chinese medicine rather than prednisone. We hope that this case report not only contributes to raising awareness of SAT related to the COVID-19 vaccine but also provides an effective remedy in addition to glucocorticoid (GC).

## Introduction

Subacute thyroiditis (SAT) is a thyroid inflammatory disease, whose pathogenesis and determinants of the clinical course were unclear for many decades. A previous viral infection is considered an SAT-triggering factor. As COVID-19 is caused by severe acute respiratory syndrome coronavirus (SARS-CoV-2) [1], it can cause various clinical manifestations, especially severe conditions such as acute respiratory distress syndrome and even death. It is noteworthy that SAT has also been reported after SARS-CoV-2 infection [2]. To deal with the global crisis, several effective vaccines have been introduced rapidly. These vaccines have shown a satisfactorily high profile of protection and safety against the disease [3]; nevertheless side effects of vaccines should be monitored and reported similarly to newly administrated drugs [4,5].

Several cases of SAT have been reported after exposure to other vaccines [6-9], but to our knowledge, only a few cases of SAT after the SARS-CoV-2 vaccination have been reported at present. These SARS-CoV-2 vaccines, after which one may develop SAT, include the adenovirus-vectored vaccine [10], inactivated SARS-CoV-2 vaccine (CoronaVac®, Sinovac Biotech, Beijing, China) [11], and messenger ribonucleic acid (mRNA) vaccine [12], however, the recombinant novel coronavirus vaccine seems to have not been mentioned before. Additionally, patients affected by SAT after vaccination were treated with conventional therapy: ibuprofen, paracetamol, or prednisone, and so on. Disappointingly, these therapies can lead to a relapse of hyperthyroidism or severe hypothyroidism [10,11]. This paper may for the first time report a case of SAT characterized by refractory hyperpyrexia post receiving a recombinant novel coronavirus vaccine (Anhui Zhifei Longcom Biopharmaceutical Co. Ltd, China), and effectively improved with herbal (traditional Chinese medicine) rather than prednisone treatment. We hope that this case report not only contributes to awareness of SAT occurring after receiving the COVID-19 vaccine but also provides an effective remedy besides the steroid hormone.

## Case presentation

A 30-year-old-male was referred from fever clinics to our outpatient clinic, complaining of high fever, neck pain, palpitations, and generalized aches, and these clinical manifestations had been going on for three weeks. There was no history of any viral or respiratory illness prior to the onset of symptoms. He had no family history of hypothyroidism or thyroiditis. He received his first dose of the vaccine created by Anhui Zhifei Longcom Biopharmaceutical Co.Ltd, China on April 25, 2021, a second dose on June 5, 2021, and his third dose on July 3, 2021. Merely two days after the third dose of the vaccine, he felt serious anterior neck pain and developed a fever. Before hospitalization, he had successively visited fever clinics twice, the department of stomatology and otolaryngological department, and been treated in vain with ibuprofen, indomethacin, lysine aspirin, and cefuroxime by his family physician. Except for the reduction of neck pain, the patient felt worse, especially with temperatures up to 39.6℃.

**Figure 1:**
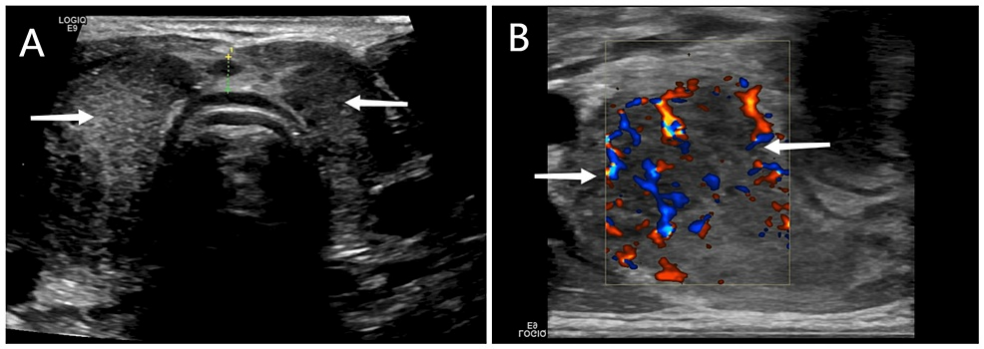
Ultrasound scan of the thyroid gland at three weeks from symptom onset. Enlarged thyroid gland with blurred margin (A), and relatively increased vascularization in ultrasound (B).

On physical examination, the heart rate was 120/min. On palpation, the thyroid gland was quite sensitive and painful, and enlarged. The polymerase chain reaction (PCR) test for SARS-CoV-2 was negative. On laboratory investigation, anti-thyroglobulin (anti-Tg) was negative, and levels of erythrocyte sedimentation rate (ESR) were high. Radioiodine uptake was decreased. Laboratory findings are summarized in Table 1. Thyroid ultrasonography (USG) showed bilateral focal hypoechoic areas but increased blood flow on Doppler USG (Figure 1). According to the diagnostic criteria of SAT [13], the diagnosis was made.

**Table 1:** Follow-up blood test results at three, eight, and 12 weeks from symptom onset after the third dose of vaccine. An improvement was seen while taking herbal decoction at 12 weeks.

Blood test	Reference range	Results at 3 weeks	Results at 8 weeks	Results at 12 weeks
Erythrocyte sedimentation rate (mm/hr)	0-15	100	34	-
Triiodothyronine(nmol/L)	0.98-2.33	3.42	2.42	1.53
Thyroxine(nmol/L)	62.68-150.84	230.98	211.200	74.46
Free triiodothyronine (pmol/L)	2.43-6.01	11.83	7.850	4.420
Free thyroxine (pmol/L	9.01-19.05	35.11	25.14	8.770
Thyroid-stimulating hormone (mIU/L)	0.35-4.94	0.0023	0.0020	8.7747
Thyroglobulin antibody (IU/ml)	<4.1	1.25	0.600	-
Thyroid receptor antibody (IU/ml)	<5.6	6.17	6.520	-

Due to the fear of recurrence of SAT and steroid dependence, the patient refused treatment with oral administration of prednisone, hence the herbs were prescribed. According to traditional Chinese medicine (TCM) theory, the disease was diagnosed as a syndrome of Qi-fen fever, and treated with a combination of huanglian jiedu decoction and baihu decoction. The prescription was administrated (one decoction per day for 12 days). Herbals, based on the theory of traditional Chinese Medicine, included huanglian (Coptis chinensis) 9g, huangqin (Scutellaria baicalensis) 12g, huangbai (Phellodendron amurense) 6g, zhizi (Gardenia) 9g, zhengshigao (raw gypsum) 25g, Zhimu (Rhizoma anemarrhenae) 10g, gancao (licorice)10g, cangzhu (Rhizoma atractylodis) 12g, fuling (Poria cocos) 15g, tianhuafen (trichosanthin) 10g, ruxiang (frankincense) 10g, moyao (myrrh)10g, inyinhua (honeysuckle) 10g, banlangen (Radix isatidis) 15g, bohe (Mentha haplocalyx) 10g, chaihu (Bupleurum) 15g. 

With the herbal treatment, the patient made a quick recovery from his illness. His complaints regressed significantly. On the second day after taking the decoction, the body temperature decreased significantly, and on the third day, the body temperature became normal, and the patient became wholly asymptomatic. On the fifth day, he was discharged on his own demand. He was followed up without treatment. At the 12th-week control visit, his thyroid function returned to essentially normal as seen above in Table 1. 

## Discussion

Subacute thyroiditis is a thyroid inflammatory disease, whose clinical course was demonstrated to evolve. The natural history of SAT persists even for months, though it is often considered to be a self-healing disease [14]. Signs and symptoms of SAT are non-characteristic, hence despite the high availability of diagnostic tools, including laboratory markers and US examination, the diagnosis of SAT is still frequently delayed [15]. The patient often visits many physicians, starting from general practitioners (GPs) through laryngologists and other specialists, before being finally diagnosed with SAT. Due to misdiagnosis of infection, antibiotics are unnecessarily administered in nearly 50% of SAT patients, and the abuse of antibiotics has led to antibiotic resistance. At present, SAT after the COVID-19 vaccine is seldom reported, which is probably because of ignorance and misdiagnosis. Thus, educational programs, especially those directed at GPs should be provided.

The treatment of SAT is also controversial. Patients with moderate-to-severe SAT symptoms are conventionally treated with 30 mg/d prednisolone [16]. A recurrent course of SAT and steroid dependence are key disadvantages of SAT treatment. The rate of SAT recurrence is rather high, despite proper diagnosis and treatment, and varies between studies from a few to over 20% [17,18]. Until recently, the cause of SAT recurrences was unknown. It is difficult to find a balance between the risk of recurrence during glucocorticoid (GC) dose tapering and the risk of complications of long-term GC therapy. Consequently, other novel therapies require further exploration. 

We report a seemingly rare case of SAT characterized by sustained high fever in a 30-year-old male without a history of autoimmune diseases, with symptoms onset two days after the third dose of the recombinant novel coronavirus vaccine. The patient did not respond well to nonsteroid anti-inflammatory drug treatment over three weeks and refused prednisone for fear of SAT recurrence and steroid dependence. It is noteworthy that he made a good recovery from the disease, with traditional Chinese medicine treatment rather than prednisone. The herbal prescription is a combination of huanglian jiedu decoction and baihu decoction, and each of them is a classical herbal formula with safety and effectiveness in the treatment of febrile Disease [19,20].

According to the herbal treatment for SAT following COVID-19 vaccination, we infer that SAT belongs to an autoimmune disorder, and herbal decoction worked by means of immunomodulation instead of immunosuppression. By the logical extension of this point, the herbal decoction may work in SARS-CoV-2 vaccination-related autoimmune diseases, and autoimmune hepatitis [21], for instance. Because of the same immunogenicity between SARS-CoV-2 and the vaccine, they can trigger a similar immune response and cause analogous clinical manifestations. As is reported, SAT occurred not only after SARS-CoV-2 infection [2] but also following SARS-CoV-2 vaccination [10-12]. Hence the herbal decoction probably effectively treats SARS-CoV-2 infection as well.

In general, the safety, effectiveness, and relevant mechanism of the treatment need further study.

## Conclusions

In conclusion, although the natural course of SAT is believed to be often self-limiting, patients frequently suffer from severe symptoms which persist even for months and rarely resolve only after glucocorticoid (GC) treatment. Nevertheless, our patient affected by SAT after the COVID-19 vaccine had a quick recovery after taking a Chinese herbal decoction rather than prednisone, which can not entirely exclude the possibility of spontaneous recovery. So It is worth further studying whether traditional Chinese medicine provides a new supplementary means for the treatment of SAT.
